# Commercially Available Mobile Apps for Caregivers of People With Alzheimer Disease or Other Related Dementias: Systematic Search

**DOI:** 10.2196/12274

**Published:** 2018-12-07

**Authors:** Lori Wozney, Luciane M Freitas de Souza, Emily Kervin, Francine Queluz, Patrick J McGrath, Janice Keefe

**Affiliations:** 1 IWK Health Centre Centre for Research in Family Health Halifax, NS Canada; 2 Nova Scotia Health Authority Dartmouth, NS Canada; 3 Nova Scotia Centre on Aging Mount Saint Vincent University Bedford, NS Canada; 4 São Francisco University Campinas Brazil; 5 Department of Family Studies and Gerontology Mount Saint Vincent University Bedford, NS Canada

**Keywords:** alzheimer and other related dementias, apps, caregivers, eHealth, mobile phone

## Abstract

**Background:**

More than 15 million Americans provide unpaid care for persons with Alzheimer disease or other related dementias (ADRD). While there is good evidence to suggest that caregivers benefit from psychosocial interventions, these have primarily been delivered via face-to-face individual or group format. Alternatively, offering electronic health (eHealth) interventions may assist caregivers in providing quality care while remaining in good health. Research to date has generated little knowledge about what app features support ADRD caregivers’ behavioral changes and how developers might optimize features over the long term.

**Objective:**

There is an evident knowledge gap in the current landscape of commercially available apps, their integration of behavioral techniques, content focus, and compliance with usability recommendations. This paper systematically reviews and inventories the apps caregivers might typically be exposed to and determines the support integrated into the apps and their functionality for older adults.

**Methods:**

The search strategy was designed to mimic typical Web-based health information-seeking behavior for adults. Apps were included based on their explicit focus on ADRD caregiver knowledge and skill improvement. Two coders with expertise in behavioral interventions and eHealth pilot-tested the data extraction. One coder retained app characteristics and design features. Techniques used to promote change were determined, and 2 questions from the Mobile App Rating Scale were used to assess the app credibility and evidence base. Content topics were evaluated using a thematic framing technique, and each app was assessed using a usability heuristic checklist.

**Results:**

The search results generated 18 unique apps that met the inclusion criteria. Some apps were unavailable, and only 8 unique apps were reviewed. Of the 8, 7 (88%) apps did not state which scientific orientation was followed to develop their content. None of the apps made clinical claims of improving caregivers’ and care recipients’ overall health. All apps relied on textual information to disseminate their contents. None of the apps was trialed and evidence based. Apps included on average 7 out of 10 behavioral change techniques, 5 out of 10 C.A.R.E. (Caregivers, Aspirations, Realities, and Expectations) features, and 10 out of 18 features on the usability heuristics checklist.

**Conclusions:**

Our findings suggest that caregivers are likely to discover apps that are not actually accessible and have low or no evidence base. Apps were found to be largely static, text-based informational resources, and few supported behaviors needed to maintain caregivers’ health. While apps may be providing a high volume of information, caregivers must still navigate what resources they need with limited guidance. Finally, the commercial marketplace is addressing some of the major usability elements, but many design elements are not addressed.

## Introduction

### Background Rationale

More than 15 million Americans provide unpaid care for persons who have reduced abilities due to Alzheimer disease or other related dementias (ADRD) [1], ranging from activities of daily living, personal care needs, shopping, and transportation. It is estimated that the 18.1 billion hours of care provided by family members or others represents a contribution of US $221.3 billion (in 2015) in unpaid support [2]. ADRD caregivers report substantial financial, emotional, and physical difficulties caring for another person [3]. Reportedly, 59% of these caregivers report that the emotional stress of caregiving is high or very high [4]. Approximately 40% report depressive symptoms, compared with 5%-17% of similar noncaregivers [5].

To help alleviate the burden associated with caregiving, health professionals, researchers, and other agencies recommend that ADRD caregivers seek support and assistance from family and friends and organize support groups [6]. While there is good evidence to suggest that caregivers benefit from psychosocial interventions, these have primarily been delivered via face-to-face individual or group format. Electronic health (eHealth) is of growing interest in expanding convenient access to information and support services. Offering eHealth to caregivers may assist them in providing quality care while remaining in good health [7].

A 2018 systematic review of eHealth interventions for family carers of people with long-term illness found that 46% (33/72) of the included studies involved ADRD caregivers [8]. The reviewer concluded that eHealth interventions for this population are “becoming more popular, and are generally perceived as acceptable, desirable and helpful” [8]. Most of the interventions in their review involved a Web-based platform with electronic or phone-based coaching support. There is evidence that caregivers may be open to lower-intensity mobile apps (mobile health) as an intervention delivery method. In 2016, 40% of caregivers reported using an app to help them manage their caregiving tasks. Caregivers aged 18-24 years are 12% of the caregiver population and account for half of the app users [9]. While older adults are slower to adopt new technologies than younger adults, they are open to using technologies that appear to have value, for example, in maintaining their quality of life [10,11].

The academic literature around mobile health for caregivers is growing and evolving somewhat analogously to the proliferation of mobile apps available in the commercial marketplace. While systematic reviews provide a snapshot of the evidence base, they do not capture the range of apps ADRD caregivers can access and download. Studies have shown that 80% of adult app downloaders rely on a rather simple “take the first” decision-making heuristic. Once they have an idea of the type of app they “need,” they pick the first one that has been well rated or ranked [12]. This rating of “top apps” is often presented through unfiltered and nonmoderated patient experience blogs, chat rooms, or testimonials from friends and family members. While the value of peer-approved resources is important, there has been concern that if unmoderated, they may expose caregivers to aggressive marketing or even unintentionally promote health behaviors that are not evidence informed [13].

Generally speaking, health apps contain low levels of behavioral change support or are not adequately designed for long-term behavioral changes [14,15]. These apps function largely as mobile information resources and do little to prompt self-care actions, lifestyle, and communication behaviors needed to successfully navigate caregiving over the long term. The complex process of family caregiving may last >20 years, suggesting that a variety of caregiver interventions are needed to support the care recipient and avoid a deleterious and compounding impact on caregivers’ health [16]. Research to date has generated little knowledge about what app features support ADRD caregivers’ behavioral changes and how developers might optimize features for adaptive and flexible behavioral skills over the long term.

Recognition of caregivers evolving needs is also underscored in recent research showing older adults have specific usability needs that influence interaction with mobile apps, but the scarce implementation of usability design guidelines for older adults has been reported [17]. Generally, the current literature suggests that although older adults are open to using technology, there may be age-related (eg, cognitive decline) as well as technology-related (eg, interface usability), barriers. As 19% of caregivers are >65 years of age [18], apps in development now must be designed with these barriers in mind. Organizations responsible for setting usability standards for eHealth tools are beginning to recommend changes to design features to make them more accessible to older adults [19], such as larger font size, no use of scrolling, use of tactile feedback (vibration), low-frequency spectrum sounds, lengthening sound signals, avoiding monochromatic color schemes, etc. Validated usability heuristics tailored for technologies involving older adults are lacking, so effort must be taekn to testing and refining emerging ways of evaluating mobile apps for ADRD caregivers [20-22].

### Research Aims and Questions

These converging issues and trends highlight a knowledge gap in the current landscape of commercially available apps, their integration of behavioral techniques, content focus, and compliance with usability recommendations. A minimum starting point for improving the design of apps for this population is to systematically review and inventory the apps caregivers might typically be exposed to, not just those studied and reported on in the academic literature. The review is guided by the following questions:

1. What are the main features and functionality of apps for caregivers of people with dementia?
2. What types of behavioral techniques are integrated into these apps?
3. What is the evidence base of apps caregivers are likely to find out about on the Web?
4. Which caregiver needs are addressed in these apps?
5. Do the apps comply with recommended usability features for older adults?

## Methods

### Search Strategy

The search strategy was designed to mimic typical Web-based health information-seeking behavior for adults as follows: (1) we chose Google, the most used search engine by adults for health information [23]; (2) we used 5 keywords per search without applying search modifiers typically used in academic reviews (eg, Boolean operators like “OR”and“AND”) as these are not commonly used by the public; (3) we conservatively reviewed the first 2 pages of search results (40 results per search) as research suggests 91% of searchers do not go past the first page of results [24]; and (4) for each search result, we limited deep navigation and only identified apps that were mentioned within 5 clicks (submenus, secondary pages, and drop-down menus) as typical users normally only proceed through 3 layers before leaving a site [25]. Overall, 5 unique searches were conducted involving combinations of keywords for the population of interest (“caregiver,” “caretaker,” “carer,” and “family”); health condition (“dementia” and “Alzheimer’s”); and technology modality (“app” and “mobile phone”). Searchers signed out of their personal Google account before conducting the search. To reduce the likelihood of locating apps that are no longer available, we filtered our search to content generated within the last 12 months. As search engine results are updated and optimized routinely, searches were conducted on the same day. Additionally, the searches were performed on a designated device and the same network to obtain consistent search results and avoid deviations by personalized search results. Searches were conducted in April 2018.

### Inclusion Criteria

**Figure 1:**
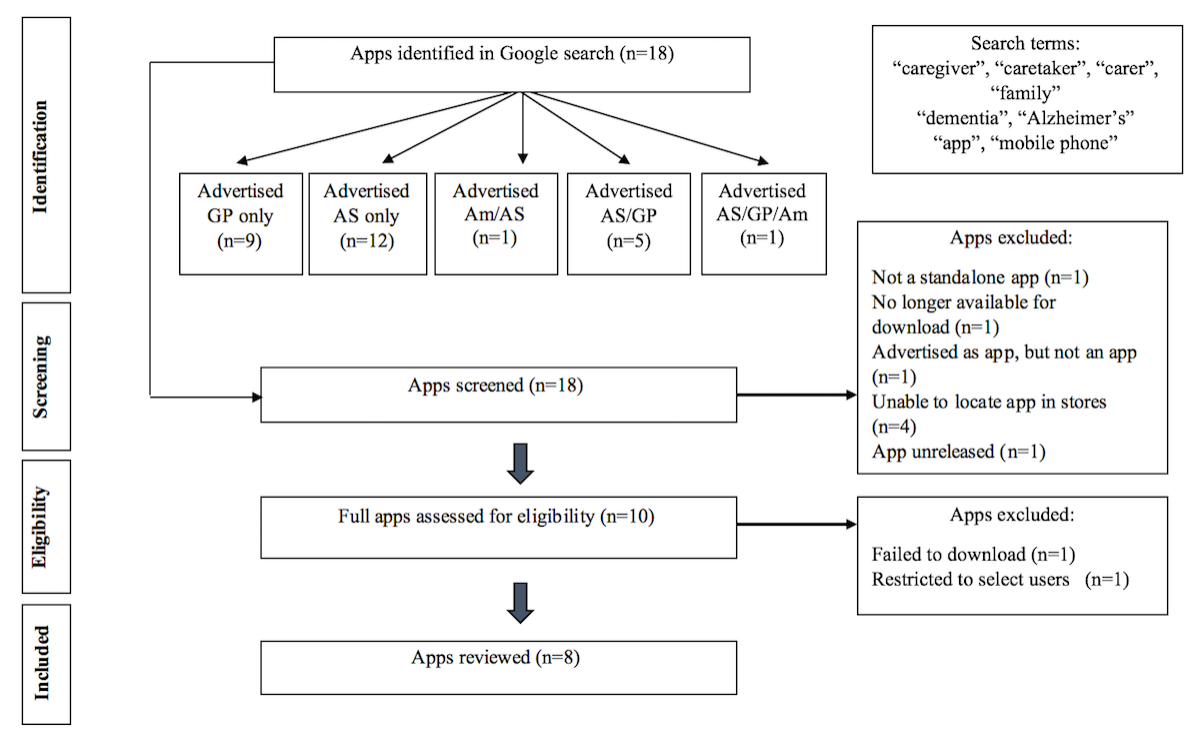
Flowchart of the selection process for apps included in the review. GP: Google Play; AS: Apple Store; Am: Advertised Amazon.

The following criteria were applied for app inclusion: (1) apps focused explicitly on improving caregivers’ knowledge or skills and (2) apps available in English. We did not exclude apps based on price, operating system (iOS, Android, Amazon, etc); popularity; or research evidence base. We excluded apps that (1) were not applicable to caregivers of people with dementia (eg, specifically for caregivers of people with cancer); apps where the target user was the care recipient and not the caregiver (eg, memory skills training app); apps where the caregiver was only tracking a single component of a care recipients’ routine (ie, medication reminder); apps that were not standalone (eg, multicomponent training program with a proprietary companion app); app that were related to generic stress, anxiety, or depression (ie, not tailored to caregivers); and apps that were no longer available for download (as of April 2018). The selection process for apps included in this study is summarized in Figure 1.

### Data Extraction

App store descriptions were scanned first to extract as much information as possible. One author (LMFdS) then downloaded each app and used it for approximately 2 days to familiarize with the features and functionality. If the app was available in multiple stores, we downloaded the one with the most recent update. Each app was navigated from the presentation screen to each menu component. The data extraction form was pilot-tested independently by 2 coders who have expertise in behavioral interventions and eHealth design (LMFdS and EK). Disagreement and discrepancies were resolved through discussion and revision of coding instructions. Data extraction was undertaken by a coder (LMFdS) for the remainder of apps with concerns and issues discussed and resolved through collaborative consensus.

For descriptive purposes, *app characteristics*, including name, identification number (ID), version, producer, price, operating system, privacy policy statement, number of downloads, and year of the last update, were retained. We extracted information on *design features*, including the use of multimedia; social interaction features; feedback and reminders; use of a persona, guide, or navigator; and instructional design elements (ie, timing, sequence, and structure of information). The presence or absence of *techniques used to promote change* was categorized using the Theoretical Domains Framework (TDF). TDF was derived from a synthesis of 33 psychological theories and 128 key theoretical constructs relevant for behavioral change [26]. It is widely used in behavioral health-related implementation and intervention research. In addition to behavioral change techniques (BCTs), we also recorded if the app endorsed a particular therapeutic (eg, cognitive behavioral therapy, behavioral activation) or theoretical approach. Two questions from the Mobile App Rating Scale [27] were used to assess app *credibility* and *evidence base.* To assess the *content topics* that each app addressed, we used the 10 C.A.R.E. Tool domains as a thematic organizing framework [28]. The C.A.R.E. Tool was developed with Health Canada as a validated psychosocial assessment tool for caregivers needs [29]. Finally, each app was assessed using a modified mobile app usability heuristic checklist for older adults proposed by Silva et al [30]. The heuristic is a composite of general and older adult-specific design recommendations and contains 18 selected items to analyze if each app considers age-related issues regarding cognition, content, dexterity, navigation, perception, and visual design. This guideline-based approach of usability testing is in accordance with the proposals of Nielsen [31].

## Results

### Principal Results

A total of 400 Google search results (10 searches x 40 results per search) returned a variety of results, including blog posts, app store advertisements, news articles, YouTube videos, and organizational websites. Close reading of these results generated a list of 18 unique app names that met the inclusion criteria. We noted 4 apps (ie, Elder411; Caregivers in the community, Small circles, and Silverline) in the search results but could not locate these in iTunes, Google Play, or Amazon apps. Several apps were referenced or cited multiple times across different search sites. A number of apps were advertised as apps for caregivers of people with dementia but were either a social network or an app to pay for health care and well-being services (eg, Huddol and WEMA Life). One app (eg, eCare) was restricted to select users only and other failed to be downloaded (eg, CaringBridge). In total, 8 unique apps were included in this review.

### General Characteristics of the Selected Apps

Of the 8 apps fully reviewed, 2 were paid. The average price of paid apps was Can $2.49 (before tax). The majority of apps (n=5) stated their privacy policy though it was unclear how clients’ data are shared with third parties. In total, 2 apps did not report a privacy statement and 1 stated that clients’ information is not shared with third parties. Moreover, 3 apps were updated in 2018, 3 in 2016, and 2 in 2015. All apps were available for download on a tablet. Notably, 1 app is developed by a Canadian organization and has its content available in English and French. A total of 6 apps targeted caregivers of people with Alzheimer and other related dementias, while 2 were generic to all caregivers. Table 1 summarizes general information of the reviewed apps.

### Main Features and Functionality of the Selected Apps

#### Content and Educational Features

In total, 7 apps (7/8, 88%) did not state which scientific orientation was followed to develop their content and 1 (1/8, 13%) app reported using “therapeutic reasoning” to recommend care strategies to caregivers. None of the apps made clinical claims of improving caregivers’ and care recipients’ emotional, mental, and physical health. Half of apps (n=4) presented content tailored for both the person with ADRD and caregivers, 2 focused on care strategies for the person with ADRD, 1 targeted the level of care to be provided to the person with ADRD, and 1 aimed to help caregivers to organize and share multiple care tasks (eg, send reminders of medication time, take the person with ADRD to a doctor appointment or stroll, and buy groceries) with a network of care supporters.

All apps relied on textual information to disseminate their content and allowed clients to select the content to be navigated; 4 apps included hyperlinks to organizations’ websites (eg, Alzheimer’s Association, American Association of Retired Persons, Mount Sinai Hospital/Reitman Centre, Home Instead Senior Care); 1 included 1-2-minute videos (ie, videos featured a professional caregiver assisting seniors with ADRD in a nursing home); 1 included audios; and 1 had animations. Only 1 app (Dementia Advisor) included an avatar, that is, a photo of the psychiatrist who had sanctioned the content of the app. Overall, 3 apps (3/8, 38%) drew on case scenarios and testimonials to build caregivers’ skills on how to deal with a person with ADRD. Notably, 7 apps did not recommend the timing of app use to caregivers (e.g. daily, weekly, or longer); 1 recommended daily access. Most apps (n=6) did not allow progress tracking, 1 used charts to demonstrate client’s progress, and 1 used textual feedback.

#### Social Media Features

Overall, 5 apps allowed the sharing of experiences in social media (e.g. closed app communities, Facebook groups, and Twitter); 1 had a discussion forum. A total of 3 apps (3/8, 38%) offered live help through helpline and texting and 1 (1/8, 13%) facilitated contact with professional caregivers and shared information with a physician. Moreover, 1 app included feedback messages (eg, automatic messages displayed in the app device) in their features; 3 apps sent reminders to clients inviting them to engage with the app (eg, to include more information about the caregiver’s needs, set care tasks, build own network—also called village).

#### Credibility and Evidence Base

In total, 4 apps (4/8, 50%) were developed by nongovernment organizations, and 1 of these organizations was granted funding by the Government of Canada’s Social Development Partnership Program, Children and Families Component; 3 (3/8, 38%) apps were developed by associations and centers for people with ADRD or retired persons; and 1 (1/8, 13%) was created by a commercial business. None of the apps has been trialed or, at least, has not published peer-reviewed research establishing their evidence base in terms of direct impact on caregiver outcomes.

#### Behavioral Change Technique Categories

**Figure 2:**
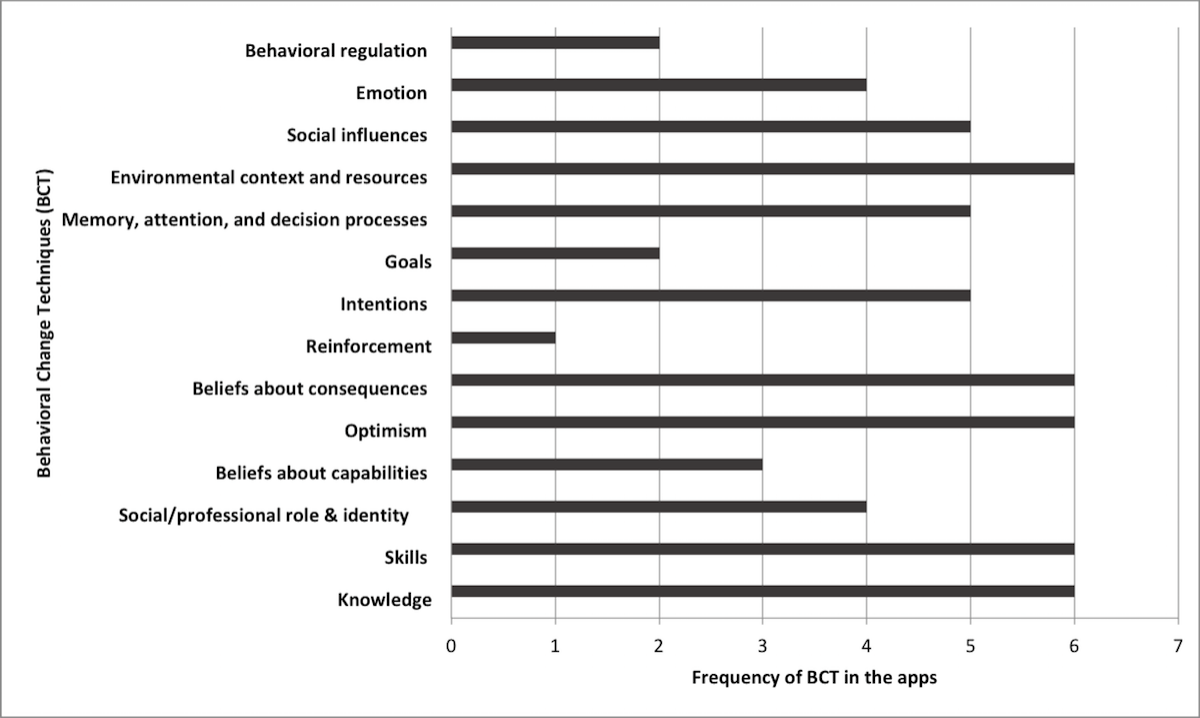
Distribution of behavioral change techniques (BCTs) across the reviewed apps.

The average number of change techniques observed per app was 7 out of 14 categories, ranging from 2 (Lotsa Helping Hands) to 12 (Balance: Alzheimer’s Caregiver and Dementia Advisor). Detailed distribution of BCTs across the apps is shown in Figure 2. The most frequent change techniques included in the apps were knowledge (n=6), skills (n=6), optimism (n=6), beliefs about consequences (n=6), and environmental context and resources (n=6). In contrast, reinforcement (n=1), goals (n=2), and behavioral regulation (n=2) were found in less frequency.

#### C.A.R.E. Features

**Figure 3:**
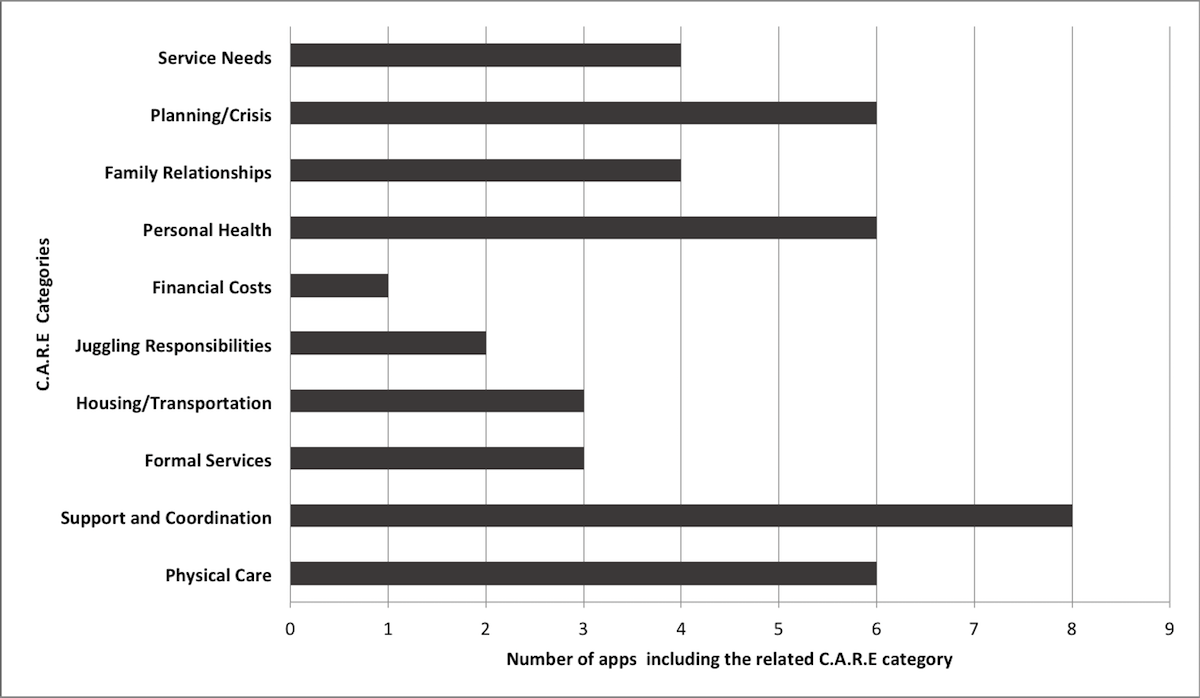
Distribution of C.A.R.E. (Caregivers, Aspirations, Realities, and Expectations) categories across the reviewed apps.

On average, apps included 5 out of 10 C.A.R.E. features, ranging from 1 (Lotsa Helping Hands) to 9 (Alzheimer’s Daily Companion). The most included features were support and coordination (n=8), physical care (n=6), personal health (n=6), planning or crisis (n=6), whereas juggling responsibilities (n=2) and financial costs (n=1) were rarely included in the apps. Distribution of C.A.R.E. features across apps is presented in Figure 3.

#### Usability Heuristics for Older Adults

Table 2 presents the distribution of usability heuristics for older adults’ features across the reviewed apps. On average, 10 out of 18 features of the modified checklist of usability heuristics for older adults were found in each app.

**Table 1:** General information of the reviewed apps.

App name	App ID number	Advertised	Developer (company name)	Operating system	Downloads (N)	Price (Can $)	Privacy statement
Alzheimer’s Daily Companion	1	Yes, Apple Store	Home Instead Senior Care	iOS and Android	500+	Free	Yes
Balance: Alzheimer’s Caregivers	2	Yes, National Alzheimer’s Centre, and Apple Store	The Hebrew Home for the Aged	iOS	NR^a^	0.99-1.39	No
Caregiver Buddy	3	Yes, Alzheimer’s Association, Apple Store, Google Play, and Amazon	Alzheimer’s Association	iOS and Android	NR	Free	Yes
Caregivers Matter^b^	4	Yes, Jewishjournal.org, Apple Store, and Google Play	Greater Lynn Senior Services (GLSS)	iOS	NR	Free	Yes
Caring Village	5	Yes, Caring.com, Google Play, and Apple Store	Caring Village	iOS and Android	1000+	Free	Yes
Dementia Advisor^c^	6	Yes, Reitman Centre, Apple Store, and Google Play	Sinai Health System-Reitman Centre	iOS	NR	Free	Yes
Dementia Caregiver Solutions^d^	7	Yes, American Seniors Housing Association, Apple Store	Lorenzo Gentile	iOS	NR	3.99	No
Lotsa Helping Hands	8	Yes, American Association of Retired Persons, Apple Store, and Google Play	Lotsa Helping Hands	Android, Web-app, and iOS	100+^e^	Free	Yes

^a^NR: not reported.

^b^App was advertised in Google Play but not found.

^c^App is also available in French.

^d^The app is related to moderate-to-severe dementia.

^e^Number of downloads on Google Play.

The most inclusive app (app 1: Alzheimer’s Daily Companion) presented 13 of 18 usability features. A feature, “content written in a language simple, clear, and adequate to older adults,” was observed in all reviewed apps. Usability principles related to cognition, dexterity, navigation, and visual design were consistently addressed across most apps.

A total of 2 apps gave instructions on how to navigate their content, presented the button “back” to facilitate app navigation, and allowed users to enlarge the font size. Only 1 app provided clear feedback when providing error messages. Overall, 3 apps (3/8, 38%) were found to use simple and meaningful icons, to make information accessible through different ways, and to aim at creating an esthetical user interface. No apps provided visual as well as tactile and auditory feedback.

**Table 2:** Distribution of usability heuristics for older adults features across the reviewed apps.

Feature		App ID number							
		1^a^	2^b^	3^c^	4^d^	5^e^	6^f^	7^g^	8^h^
**Cognition**									
	Focus on 1 task at a time instead of requiring the user to actively monitor ≥2 tasks and clearly indicate the name and status of the task at all times.	✓	✓	✓	✓	✓	✓	✓	N/A^i^
	Avoid the use of animation and fast-moving objects.	✓	✓	✓	✓	✓	N/A	✓	✓
	Aim at creating an esthetical user interface by using pictures and graphics purposefully and adequately to minimize user interface clutter and avoid extraneous details.	N/A	N/A	N/A	✓	✓	✓	N/A	N/A
**Content**									
	Give specific and clear instructions and make help and documentation available. Remember that it is better to prevent an error than to recover from it.	N/A	N/A	N/A	✓	N/A	N/A	N/A	N/A
	Provide clear feedback and when presenting error messages make them simple and easy to follow.	N/A	✓	N/A	N/A	N/A	N/A	N/A	N/A
	Write in a language that is simple, clear, and adequate to the audience.	✓	✓	✓	✓	✓	✓	✓	✓
**Dexterity**									
	Avoid pull-down menus.	✓	✓	✓	N/A	✓	✓	✓	✓
	Avoid the use of scrolling.	✓	N/A	✓	✓	✓	✓	✓	✓
	Enlarge the size of user interface elements in general; targets should be, at least, 14 mm^2^.	N/A	✓	N/A	N/A	N/A	✓	N/A	N/A
**Navigation**									
	Keep the user interface navigation structure narrow, simple, and straightforward.	✓	✓	✓	✓	✓	✓	✓	N/A
	Make sure that the “Back” button behaves predictably.	✓	N/A	N/A	N/A	N/A	✓	N/A	N/A
**Perception**									
	Provide not only visual but also tactile and auditory feedback.	N/A	N/A	N/A	N/A	N/A	N/A	N/A	N/A
	Make information accessible through different modalities.	✓	N/A	✓	N/A	N/A	✓	N/A	N/A
**Visual Design**									
	Use high-contrast color combinations of font and graphics and background to ensure readability and perceptibility; avoid using blue, green, and yellow in close proximity.	✓	N/A	N/A	✓	✓	✓	✓	✓
	Use color conservatively, limiting the maximum number of colors in use to ~4.	✓	N/A	✓	✓	✓	✓	✓	✓
	Make links and buttons clearly visible and distinguishable from other user interface elements.	✓	✓	N/A	N/A	✓	✓	✓	N/A
	Use user interface elements consistently and adhere to standards and conventions if those exist.	✓	✓	N/A	N/A	N/A	✓	✓	N/A
	Use simple and meaningful icons.	✓	✓	N/A	N/A	N/A	✓	N/A	N/A
Total features		13	10	8	9	10	14	10	6

^a^App 1: Alzheimer’s Daily Companion.

^b^App 2: Balance: Alzheimer’s Caregivers.

^c^App 3: Caregiver Buddy.

^d^App 4: Caregivers Matter.

^e^App 5: Caring Village.

^f^App 6: Dementia Advisor.

^g^App 7: Dementia Caregiver Solution.

^h^App 8: Lotsa Helping Hands.

^i^N/A: not applicable.

## Discussion

### Principal Findings

This study aimed to systematically review apps that caregivers of persons with dementia might typically encounter in the commercial marketplace. Our findings suggest that when looking for apps to support them in their role, caregivers are likely to discover apps that are not actually accessible or available in the app store or are linked to specific services or programs they cannot access. For this reason, our review provides only a snapshot of what was available at the time of our search. Even when the app store logo was present on a website, it did not ensure that the app was still available for download. This would likely be frustrating, confusing, and potentially create barriers to future health information seeking. Our review also showed that the evidence base for commercially available apps for this population is low or none at the time of our review. While the app might still be perceived as useful or helpful to caregivers, it is unclear how to manage caregivers’ expectations about if and how these apps might help given the lack of rigorous outcome monitoring.

Apps in our review were found to be largely static, text-based informational resources focusing on relaying factual information and proving common-sense tips and ideas for coping. Few of the apps emphasized goal setting, action planning, or self-monitoring, but they did not provide tailored feedback or reinforcement techniques that support lifestyle or behavioral changes needed to maintain their own health. Some of the apps did provide a Web-based community for all users of the app to connect and share, but it is unclear as to the content of these interactions, their perceived utility, or restrictions (eg, geographic location, language, etc) that might limit users’ access. Closed groups in which users can share information with a preset list of approved family members or friends were one of the more common design features. Apps employed limited multimedia outside of hyperlinks to additional textual information or websites.

As mainly informational resources, it is not surprising that, overall, apps covered significant breadth of caregiving-related topics within the C.A.R.E. (Caregivers, Aspirations, Realities, and Expectations) Tool domains. While diverse topics were covered, there was no evidence that these apps were able to tailor or personalize the informational needs to specific users, type of dementia, stage of disease, or other caregiving scenarios (eg, coresiding vs living apart). This suggests that while apps may be providing high volume of information, caregivers must still filter, navigate, and adjudicate what resources they need with limited feedback and guidance in navigation aside from key search terms or alphabetized lists of topics.

Finally, this review of usability heuristics suggests that the commercial marketplace is addressing some of the major usability elements (clear, simple language) but that many perceptual and visual design elements are not addressed. Importantly, this is an emerging field with new understandings evolving rapidly about what older adults typically need to support their interaction with mobile apps and how unique cognitive, motivational, and developmental realities of that population should be incorporated into the design.

### Limitations

Our review has some limitations. Approximating typical search strategies was based on research about how adults access information on the Web. While we conservatively reviewed more search engine results to account for divergent approaches, it is possible that caregivers might employ different or more diverse strategies and may locate or identify different apps than those we captured. Search location algorithms used by Google may have also unintentionally biased results geographically. While an adjudication process with at least 2 authors was used to find consensus on inclusion screening and app data extraction, interrater reliability or kappa was not calculated. TDF is a framework for exploring behavioral change, but we only applied it to the global domains level, which limits the level of specificity that we could speak to. Finally, we did not evaluate the accuracy or evidence base of the information being relayed within the apps—only general information about topics, design features, and usability.

### Future Directions

Our findings point to 4 key areas for innovative research and future intervention development. First, moving beyond informational apps to harnessing new functionality of apps to deliver a much more tailored and personally relevant learning experience is possible. Information is only valuable if caregivers can mobilize it to improve their quality of life. Apps that not only provide information but also help caregivers think about ways to put that information into action may support better self-management. Second, the field of gerontechnology needs to rapidly develop industry guidelines around Web design that is sensitive to the unique, visual, perceptual, cognitive, and motivational attributes of older adults that typically act as caregivers. Best practices in usability should be a priority as one size does not fit all in design for adults. Older adults need their user experience to be better understood by developers and should not be assumed to operate in the same ways as younger adults. Third, prioritizing or weighting of informational needs is an important line of future research. While apps may provide comprehensive information, not all caregivers need all of it at the same time. Learning more about when different informational needs present along the caregiver journey, which needs are more critical or impactful on the quality of life, and how different types of dementia or stage of disease make information more or less relevant is vital. Mapping of caregiver journey typologies could help ensure designers move away from information repositories toward truly personalized “just-in-time” interventions. Finally, this review highlights the importance of co-design for mobile app development. Caregivers of persons with dementia face incredibly complex informational needs. True design partnerships for mobile apps with this population should be built holistically—taking into account not just what they need but how they want to interact with the app, why and where they find value in different features, and how best to make the apps accessible and available to them.

## Abbreviations

ADRD: Alzheimer disease or other related dementias
BCT: behavioral change technique
C.A.R.E.: Caregivers, Aspirations, Realities, and Expectations
eHealth: electronic health
TDF: Theoretical Domains Framework
